# Nanopore sequencing reads improve assembly and gene annotation of the *Parochlus steinenii* genome

**DOI:** 10.1038/s41598-019-41549-8

**Published:** 2019-03-25

**Authors:** Seung Chul Shin, Hyun Kim, Jun Hyuck Lee, Han-Woo Kim, Joonho Park, Beom-Soon Choi, Sang-Choon Lee, Ji Hee Kim, Hyoungseok Lee, Sanghee Kim

**Affiliations:** 1 Unit of Polar Genomics, Korea Polar Research Institute (KOPRI), Incheon, 21990 Republic of Korea; 2 Department of Polar Sciences, University of Science and Technology, Incheon, 21990 Republic of Korea; 3 Department of Fine Chemistry, Seoul National University of Science and Technology, Seoul, 01811 Republic of Korea; 4 Phyzen Co., Ltd, Seongnam, 13558 Republic of Korea; 5 Division of Life Sciences, Korea Polar Research Institute (KOPRI), Incheon, 21990 Republic of Korea

## Abstract

*Parochlus steinenii* is a winged midge from King George Island. It is cold-tolerant and endures the harsh Antarctic winter. Previously, we reported the genome of this midge, but the genome assembly with short reads had limited contig contiguity, which reduced the completeness of the genome assembly and the annotated gene sets. Recently, assembly contiguity has been increased using nanopore technology. A number of methods for enhancing the low base quality of the assembly have been reported, including long-read (e.g. Nanopolish) or short-read (e.g. Pilon) based methods. Based on these advances, we used nanopore technologies to upgrade the draft genome sequence of *P. steinenii*. The final assembled genome was 145,366,448 bases in length. The contig number decreased from 9,132 to 162, and the N50 contig size increased from 36,946 to 1,989,550 bases. The BUSCO completeness of the assembly increased from 87.8 to 98.7%. Improved assembly statistics helped predict more genes from the draft genome of *P. steinenii*. The completeness of the predicted gene model increased from 79.5 to 92.1%, but the numbers and types of the predicted repeats were similar to those observed in the short read assembly, with the exception of long interspersed nuclear elements. In the present study, we markedly improved the *P. steinenii* genome assembly statistics using nanopore sequencing, but found that genome polishing with high-quality reads was essential for improving genome annotation. The number of genes predicted and the lengths of the genes were greater than before, and nanopore technology readily improved genome information.

## Introduction

*Parochlus steinenii* is a winged midge found on islands off the coast of Antarctica^1,2^. It is a polytypic species and is widely distributed throughout Patagonia and the Maritime Antarctic and sub-Antarctic^1^. *P. steinenii* midges from the Maritime Antarctic are more closely related to those from the sub-Antarctic than to those from Patagonia. The divergence period between midges from the Maritime Antarctic South Shetland Islands and those from sub-Antarctic South Georgia is 7.6 million years (Myr)^3^. In the maritime Antarctic, another midge, *Belgica antarctica*, occur naturally with *P. steinenii*^1^. The wingless midge, *B. antarctica* are freeze-tolerant in their larval stage, and the draft genome was recently reported^4^. However, *P. steinenii* are not freeze-tolerant but cold-tolerant^1^. This different adaption in Antarctic midges are interesting in terms of evolutionary processes within a harsh environment. Previously, we have reported the genome of the Antarctic midge *P. steinenii*^5^, but the completeness of the genome assembly was only 67.2% and the completeness of the annotated gene sets was only 70.7%. The genome completeness and gene set completeness of draft genome of *B. antarctica* is 86.4% and 86.6%, respectively. These results were due to the limited contig contiguity in the draft genome of *P. steinenii*. Recently, there have been many reports of improvements in assembly using nanopore technology^6–10^. Base-calling methods have been improved sufficiently^11,12^, thus the base quality of nanopore reads was reported to be enough for the *de novo* genome assembly^6,7,10,13^. The development of ultra-long reads up to 882 kb is only a merit of nanopore technology^8^. Various methods for improving low base quality of the assembled sequence have also been reported^10,14^. High quality reads and signal-level data of nanopore reads were used to improve the base quality of draft genome sequence^10,14^. In this study, we applied these nanopore technologies to upgrade the draft genome sequence of *P. steinenii*. Prior to a comparative analysis between Antarctic midges, we investigated the difference in annotation.

## Results and Discussion

### Oxford Nanopore Technology 1D sequencing

We obtained 2 μg of total DNA from 50 adult midges, and constructed an Oxford Nanopore Technologies (ONT) library. The total amount of final library was 930 ng of DNA (Table 1). Through ONT 1D sequencing using a single 1D flow cell, 10,970,289,711 bases were identified from 1,999,088 reads (Table 2).

**Table 1:** Library preparation.

	After DNA repair	After end repair	After ligation
PicoGreen assay (ng/μL)	16	29	62
Total amount (ng)	1,600	870	930

**Table 2:** Summary of nanopore read statistics.

	Raw data	Corrected read
Total read number	1,999,088	341,108
Total read bases (bp)	10,970,289,711	5,742,044,883
Mean read length (bp)	5487.61 (10.4)	16,986
Max length (bp)	96,705	87,202
Read length N50 (bp)	12,381	17,615
Number above 5 kbp/total length (bp)/percentage of the total reads (%)	692,507/8,819,419,598/80	340,083/5,739,314,651/100
Number above 10 kbp/total length (bp)/percentage of the total reads (%)	378,620/6,548,956,539/60	327,418/5,616,993,576/96
Number above 20 kbp/total length (bp)/percentage of the total reads (%)	101,037/2,638,003,734/24	81,947/2,110,920,760/39

kbp = kilo base pairs. The raw data were base-called using Guppy software, and Canu was used to correct the longest reads up to 40× coverage as default.

We found that 80% of all reads were longer than 5 kilo base pairs (kbp), 60% of reads were longer than 10 kbp, and 24% of reads were over 20 kbp. The longest read comprised 96,705 bases, and the reads had a mean Phred score (a measure of the quality of base identification) of over 10.4.

### *De novo* genome assembly of Illumina reads and nanopore reads

The scaffold sequence generated from ALLPATHS-LG in a previous study^5^ contained information about ambiguities within the assembly. For comparison with assemblies from nanopore reads, we removed the assembly ambiguity information, and filled the gaps in the resulting scaffolds. The final assembly using Illumina reads had a total size of 138 mega base pairs (Mbp), comprising 9,132 contigs with an N50 contig size of 36,946 and an N50 scaffold size of 176 kbp (Table 3).

**Table 3:** Genome assembly statistics.

	IR	NR
Number of scaffolds	4,127	162
Number of contigs	9,132	162
Total scaffold sequence (bp)	138,124,775	145,366,448
Total contig sequence (bp)	130,756,571	145,366,448
Length of N50 scaffold (bp)	176,193	1,989,550
Length of N50 contig (bp)	36,946	1,989,550
Max scaffold length (bp)	655,752	9,644,260
Max contig length (bp)	320,332	9,644,260

IR = the draft genome sequence assembled from the Illumina reads; NR = the draft genome sequence assembled with nanopore reads. The Illumina reads were initially assembled using ALLPATHS-LG with Illumina short reads, and gap-filled using GapFiller. The nanopore reads were assembled with nanopore reads corrected by Canu using SMARTdenovo.

Assembly of the nanopore reads was performed using the Canu-SMARTdenovo method^15^. Nanopore reads were corrected with Canu (ver. 1.1.1)^16^ before assembly, and we obtained 341,108 corrected reads with 5,742,044,883 bp (Table 2). All trimmed reads were longer than 5 kb, 96% were longer than 10 kb, and 39% were longer than 20 kb. The maximum read length was reduced to 87,202 bp. The resulting reads were assembled using SMARTdenovo^17^. The final assembled genome comprised 145,366,448 bp, the number of contigs decreased from 9,132 to 162, and the N50 contig size increased from 36,946 to 1,989,550 bp. The maximum contig size increased markedly from 320,332 to 9,644,260 bp (Table 3). The draft genome sequence assembled from nanopore reads (NR) exhibited excellent contiguity compared to that of the draft genome sequence assembled from the Illumina reads (IR).

### Genome polishing and the genome completeness of draft genome sequences

**Figure 1:**
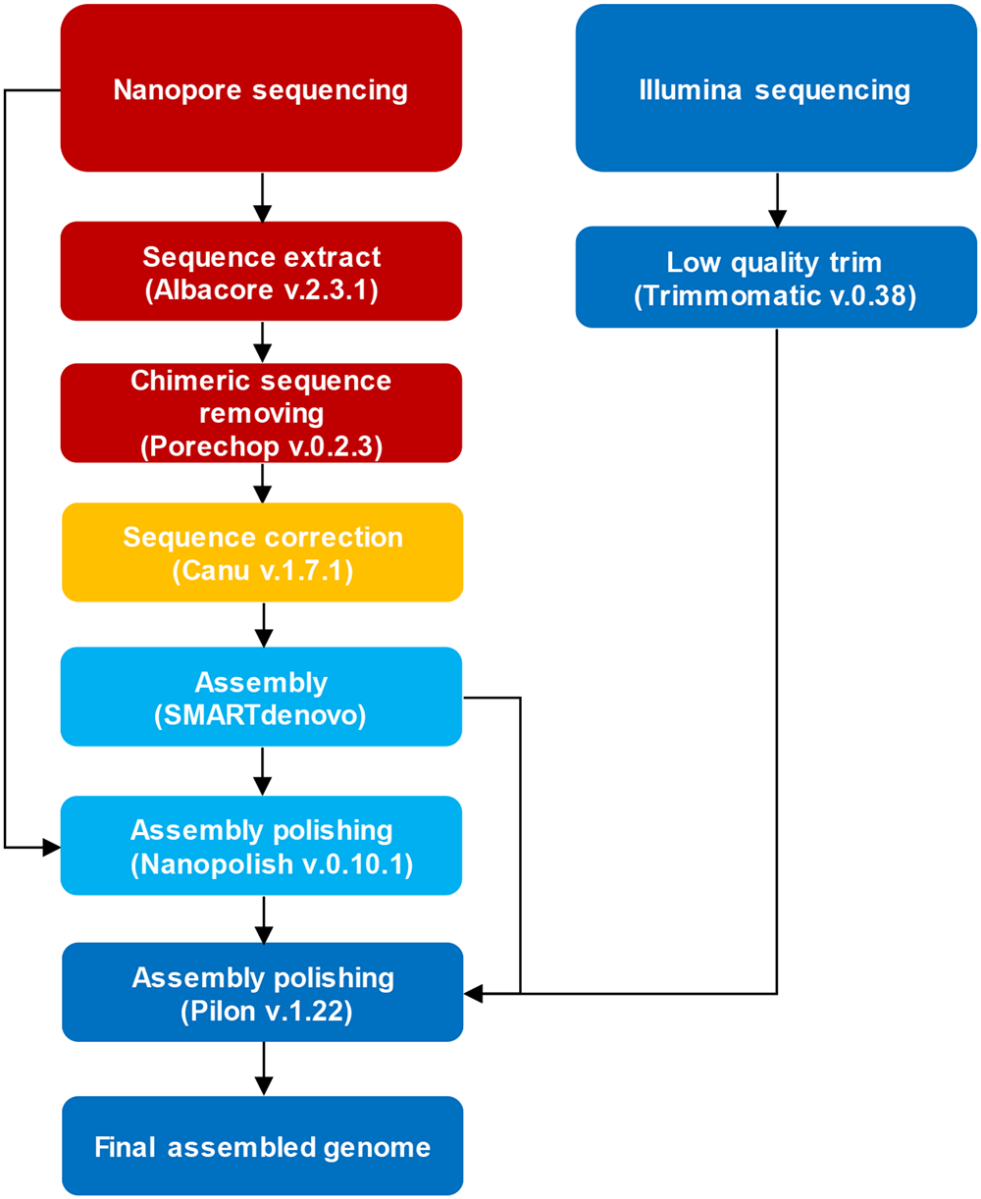
Data analysis overview. We used Albacore (ver. 2.3.1) to base-call the nanopore sequencing reads, and used Canu (ver. 1.7.1) to correct the nanopore reads. We assembled the resulting corrected reads into contigs using SMARTdenovo, and genome polishing was performed using Pilon (ver. 1.22) and Nanopolish (ver. 0.10.1).

The accuracy of draft genome sequences assembled from nanopore sequencing reads is reported to be below 98%^8^. We used two programs to improve the accuracy of the draft genome sequence (Fig. 1)^8^. First, we used Nanopolish (ver. 0.10.1)^10^, which is a software package for single-level analysis of nanopore sequencing. Nanopolish can improve the quality of the consensus sequence through signal-level data in the FAST5 files. We used the newly aligned read information about the draft assembly obtained using BWA (ver. 0.7.17)^18^ and the signal-level data to improve the quality of the consensus sequence during genome polishing^10^. Next, we used Pilon (ver. 1.22) to polish the draft assembly^14^. Pilon was developed to improve variant detection and genome assembly. It uses high-quality reads such as an Illumina reads to correct draft assemblies constructed from relatively low-quality reads^8,14^. After genome polishing of NR, the identities between IR and NR increased from 0.53 to 0.79% (Table 4). However, the maximum identity was below 99%. This may have been due to heterogeneity and variation in the DNA samples, which were obtained at different times, even from the same site.

**Table 4:** Summary of genome polishing.

Assembly	Assembler	Genome polishing	Identity between aligned regions
IR	ALLPATHS-LG	None	
NR	SMARTdenovo	None	98.15%
NR + np	SMARTdenovo	Nanopolish	98.68%
NR + pl	SMARTdenovo	Pilon	98.90%
NR + np + pl	SMARTdenovo	Nanopolish + Pilon	98.93%
NR + np + pl × 2	SMARTdenovo	Nanopolish + Pilon × 2	**98.94%**

IR = the draft genome sequence assembled from the Illumina reads; NR = the draft genome sequence assembled from nanopore reads. The identity between aligned regions values were calculated using nucmer and dnadiff. The bold characters indicate the best identity.

**Figure 2:**
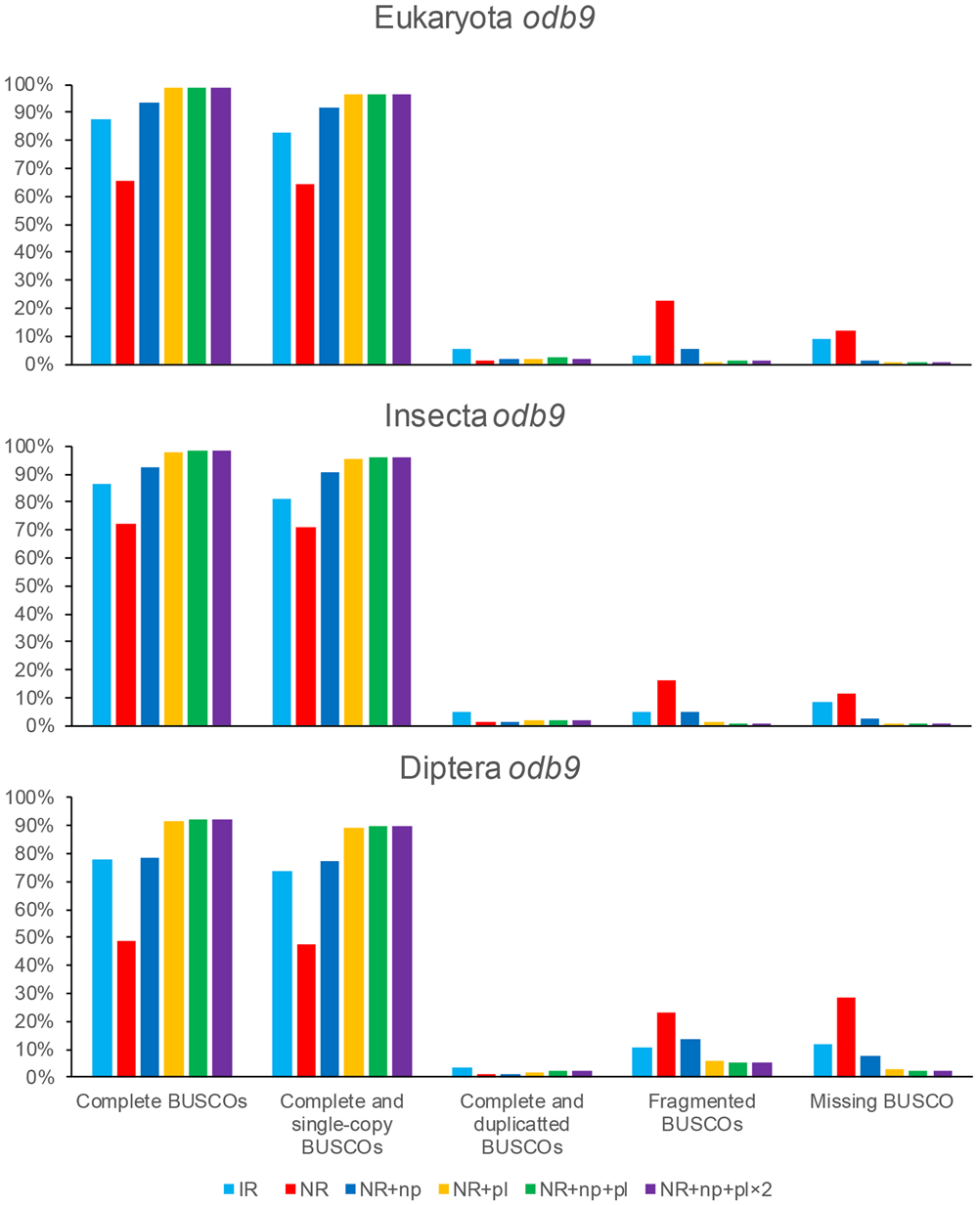
Benchmarking universal single-copy orthologs (BUSCO) analysis of draft genome sequences. The genome completeness values of six draft genome sequences were calculated using BUSCO against Eukaryota *odb9*, Insecta *odb9*, and Diptera *odb9*. Before genome polishing, the low-quality NR reduced the completeness of the genome and increased the number of “Fragmented BUSCOSs” and “Missing BUSCOs.” Genome polishing of the NR improved the completeness of the genome, and the use of Illumina reads markedly improved genome polishing with signal-level data in BUSCO analysis.

The genome completeness of the draft genome sequences was validated using benchmarking universal single-copy orthologs (BUSCO; ver. 3)^19,20^. We conducted BUSCO analyses against Eukaryota, Insecta, and Diptera datasets (Fig. 2 and Table 5). Although the contiguity of the NR markedly improved, BUSCO completeness assessments for the genome were lower than those of the IR. As BUSCO estimates the genome completeness by gene annotation using Augustus with BUSCO group consensus sequences, the bases exhibiting low quality in the NR may decrease the rate of gene annotation and lower the rates of BUSCO completeness assessments for the genome. Given this, we could identify that genome polishing improving the accuracy of base qualities increased BUSCO completeness assessment for the genome of the NR (Tables 4 and 5). Although the identity did not increase dramatically after genome polishing, the genome completeness assessment of the NR obtained using Nanopolish with signal-level data (NR + np) increased to a level similar to that of the IR. Nanopolish improved the genome completeness assessment, but the effect was less than that of genome polishing using Illumina reads. Genome polishing with Pilon using Illumina reads (NR + pl, NR + np + pl, and NR + np + pl × 2) increased completeness values of NRs to more than 98.7% in the BUSCO analysis against Eukaryota *odb9*, to 97.9% against Insecta *odb9*, and to 91.3% against Diptera *odb9* (Fig. 2). Genome polishing using Pilon alone markedly increased the genome completeness assessment of the NRs.

**Table 5:** BUSCO completeness assessments for genomes.

Database	Assemblies and genome polishing	Complete BUSCOs	Duplicated BUSCOs	Fragmented BUSCOs	Missing BUSCOs	Total BUSCO groups searched orthologs
Eukaryota *odb9*	IR	87.8%	5.3%	3.0%	9.2%	303
	NR	67.7%	1.3%	22.4%	12.2%	303
	NR + np	93.4%	1.7%	5.3%	1.3%	303
	**NR + pl**	**98.7%**	**2.0%**	**0.7%**	**0.7%**	303
	NR + np + pl	98.7%	2.3%	1.0%	0.3%	303
	NR + np + pl × 2	98.7%	2.0%	1.0%	0.3%	303
Insecta *odb9*	IR	86.6%	5.2%	5.1%	8.3%	1,658
	NR	72.2%	1.4%	16.2%	11.6%	1,658
	NR + np	92.3%	1.4%	4.8%	2.9%	1,658
	NR + pl	97.9%	2.2%	1.4%	0.7%	1,658
	**NR + np + pl**	**98.4%**	**2.2%**	**0.8%**	**0.8%**	1,658
	NR + np + pl × 2	98.3%	2.2%	0.8%	0.8%	1,658
Diptera *odb9*	IR	77.7%	3.7%	10.6%	11.7%	2,799
	NR	48.8%	1.1%	22.9%	28.3%	2,799
	NR + np	78.5%	1.3%	13.6%	8.0%	2,799
	NR + pl	91.3%	2.0%	6.0%	2.7%	2,799
	**NR + np + pl**	**92.0%**	**2.3%**	**5.5%**	**2.5%**	2,799
	NR + np + pl × 2	92.0%	2.3%	5.5%	2.6%	2,799

IR = the draft genome sequence assembled from the Illumina reads; NR = the draft genome sequence assembled from nanopore reads. The bold characters indicate the best statistics of genome completeness assessment using BUSCO.

### Repeat analysis and non-coding RNA

The total coverage of repeat sequences in *P. steinenii* ranged from 6.74 to 11.89% of the total contig length (Table 6). Almost all statistics for repeats were similar among the draft genome sequences (Table 6); however, the number and the total length of masked interspersed repeats increased in the NR, and those of predicted long interspersed nuclear elements (LINEs) and unclassified repeat among the interspersed repeats increased markedly (Table 7). The total length of non-LTR retrotransposons comprise long interspersed nuclear elements (LINEs), and short interspersed nuclear elements (SINEs) also increased. The number of predicted tRNAs ranged from 151 to 172 (Table 6).

**Table 6:** Major repetitive content and tRNAs.

	IR	NR	NR + np	NR + pl	NR + np + pl	NR + np + pl × 2
Interspersed repeats	7,639,658 (26,042)	14,540,409 (32,830)	14,662,939 (33,009)	14,547,597 (32,603)	14,751,532 (33,069)	14,754,452 (33,063)
Simple repeats	1,165,508	1,225,771	1,208,581	1,219,354	1,217,748	1,218,017
Low complexity	438,219	433,317	430,197	430,290	430,938	432,152
tRNA	13,137 (172)	11,529 (151)	11,306 (151)	11,411 (153)	11,328 (152)	11,328 (152)

IR = the draft genome sequence assembled from the Illumina reads; NR = the draft genome sequence assembled from nanopore reads. The total lengths of the repeats and tRNAs were calculated using RepeatMasker^30^ and tRNAscan-SE^35^, respectively, and the number of elements is given in parentheses.

**Table 7:** Statistics of interspersed repeats contents.

	IR	NR	NR + np	NR + pl	NR + np + pl	NR + np + pl × 2
SINE	68,267 (88)	100,381 (97)	101,304 (97)	101,569 (98)	102,052 (98)	102,006 (98)
LINE	524,538 (1,291)	942,262 (1,600)	959,395 (1,614)	949,814 (1,593)	963,093 (1,610)	963,118 (1,609)
LTR	279,691 (568)	1,595,603 (1,087)	1,600,930 (1,102)	1,596,730 (1,097)	1,604,972 (1,108)	1,605,234 (1,104)
DNA	267,157 (1,038)	370,673 (1,234)	375,621 (1,250)	375886 (1,239)	378,520 (1,253)	378,616 (1,251)
Unclassified	6,500,005 (23,057)	11,531,490 (28,812)	11,625,779 (28,946)	11,523,598 (28,576)	11,702,895 (29,000)	11,705,478 (29,001)
Total interspersed repeats	7,639,658	14,540,409	14,662,939	14,547,597	14,751,532	14,754,452

IR = the draft genome sequence assembled from the Illumina reads; NR = the draft genome sequence assembled from nanopore reads. The total lengths of repeats and tRNAs were calculated using RepeatMasker, and the number of elements is given in parentheses. Long terminal repeats (LTRs) are retrotransposons, and non-LTR retrotransposons comprise long interspersed nuclear elements (LINEs) and short interspersed nuclear elements (SINEs).

### Gene annotation and gene set completeness of draft genome sequences

As reported in Table 8, 11,690 genes were predicted in the IR. The number of genes in NRs (NR + np, NR + pl, NR + np + pl, and NR + np + pl × 2) was predicted to be similar. Except for the NR, the number of genes ranged from 11,690 to 12,074. A relatively large number of genes (16,956) was predicted in the NR compared to the other draft genome sequences, whereas the total length of the gene regions was smaller than in the others sequences. The total length of the gene regions increased in NRs (NR + np, NR + pl, NR + np + pl, and NR + np + pl × 2) after genome polishing, but the total lengths of the coding sequence and gene regions did not increase compared with the total length of the gene regions in NR + np. Instead, the total lengths of intron and untranslated regions (UTRs) increased. In the NRs polished using Pilon (NR + pl, NR + np + pl, and NR + np + pl × 2), the total lengths of the exons, coding sequences (CDSs), and introns increased, and the total lengths of the 5′-UTR and 3′-UTR regions were similar to those of the IR (Table 8).

**Table 8:** Summary of MAKER2 annotation.

		IR	NR	NR + np	NR + pl	NR + np + pl	NR + np + pl × 2
gene	number^a^	11690	**16956**	11971	12074	11938	11935
	length^b^	51671609 (4420.2)	47351244 (2792.6)	59346690 (4957.5)	59414543 (4920.9)	**60270059** (5048.6)	59995550 (5026.9)
CDS	number	90583 (7.7)	72775 (4.3)	104540 (8.7)	103425 (8.6)	**104125** (8.7)	103928 (8.7)
	Length	19208721 (1643.2)	11638566 (686.4)	18935550 (1581.8)	**21849837** (1809.7)	21627003 (1811.6)	21615393 (1811.1)
exon	number	91886 (7.9)	87307 (5.1)	**107462** (9.0)	104883 (8.7)	105527 (8.8)	105335 (8.8)
	Length	21402569 (1830.8)	20493668 (1208.6)	21782057 (1819.6)	**24119815** (1997.7)	23810842 (1994.5)	23809534 (1994.9)
intron	number	80196 (6.9)	70351 (4.1)	95491 (8.0)	92809 (7.7)	**93589** (7.8)	93400 (7.8)
	Length	30269040 (2589.32)	26857576 (1584.0)	**37564633** (3138.0)	35294728 (2923.2)	36459217 (3054.0)	36186016 (3031.9)
5′-UTR	number	4514 (1.3)	**14085** (2.1)	5399 (1.5)	4627 (1.3)	4537 (1.3)	4581 (1.3)
	Length	471401 (134.4)	**3544608** (524.0)	807804 (219.2)	484738 (136.2)	484557 (138.5)	484432 (136.7)
3′-UTR	number	4117 (1.1)	**13975** (2.1)	5049 (1.3)	4394 (1.1)	4255 (1.1)	4274 (1.1)
	Length	1722447 (447.0)	**5310494** (783.5)	2038703 (525.4)	1785240 (441.6)	1699282 (432.8)	1709709 (433.5)

CDS = coding sequence; IR = the draft genome sequence assembled from the Illumina reads; NR = the draft genome sequence assembled from nanopore reads; UTR = untranslated region. The numbers and total lengths of the genes, CDSs, exons, introns, and UTRs were calculated from a GFF3 file generated by MAKER2^21,36^, and the unit averages are given in parentheses. In each row, the best results are shown in bold.

^a^Denotes the number of elements.

^b^Denotes the total length of the elements.

**Figure 3:**
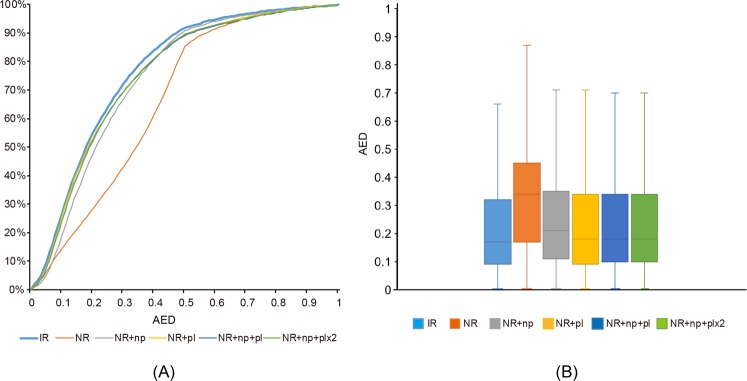
Annotation edit distance (AED) metric for controlling the quality of annotation for the final gene predictions of the six drafts of the genome sequences. (**A**) The cumulative AED distribution for all six draft genomes. (**B**) Box plot of AED scores for all six draft genomes.

Annotation edit distance (AED) values of annotated genes lie between 0 and 1; if the alignment evidence matches the annotated gene exactly, the AED value is 0; if there is no supporting evidence, the AED value is 1^21^. Figure 3 comprises a plot of the cumulative distribution of the AED values for each assembly and a box plot of the AED scores. The AED distribution of NR + np was shifted slightly toward lower AED values relative to the IR below 0.5, and those of the NR were shifted toward much lower AED values than NR + np. The AED distribution of the IR and the NRs polished using Pilon (NR + pl, NR + np + pl, and NR + np + pl × 2) had similar cumulative distributions of AED below 0.2, but those of the NRs were shifted slightly to lower AED values relative to the IR from an AED value of 0.2 (Fig. 3a). In the box plot, the 25th percentile, the 75th percentile, and the median showed that the annotated gene quality of the NRs polished with Illumina reads (NR + pl, NR + np + pl, and NR + np + pl × 2) did not increase markedly compared with that of the IR (Fig. 3b).

**Figure 4:**
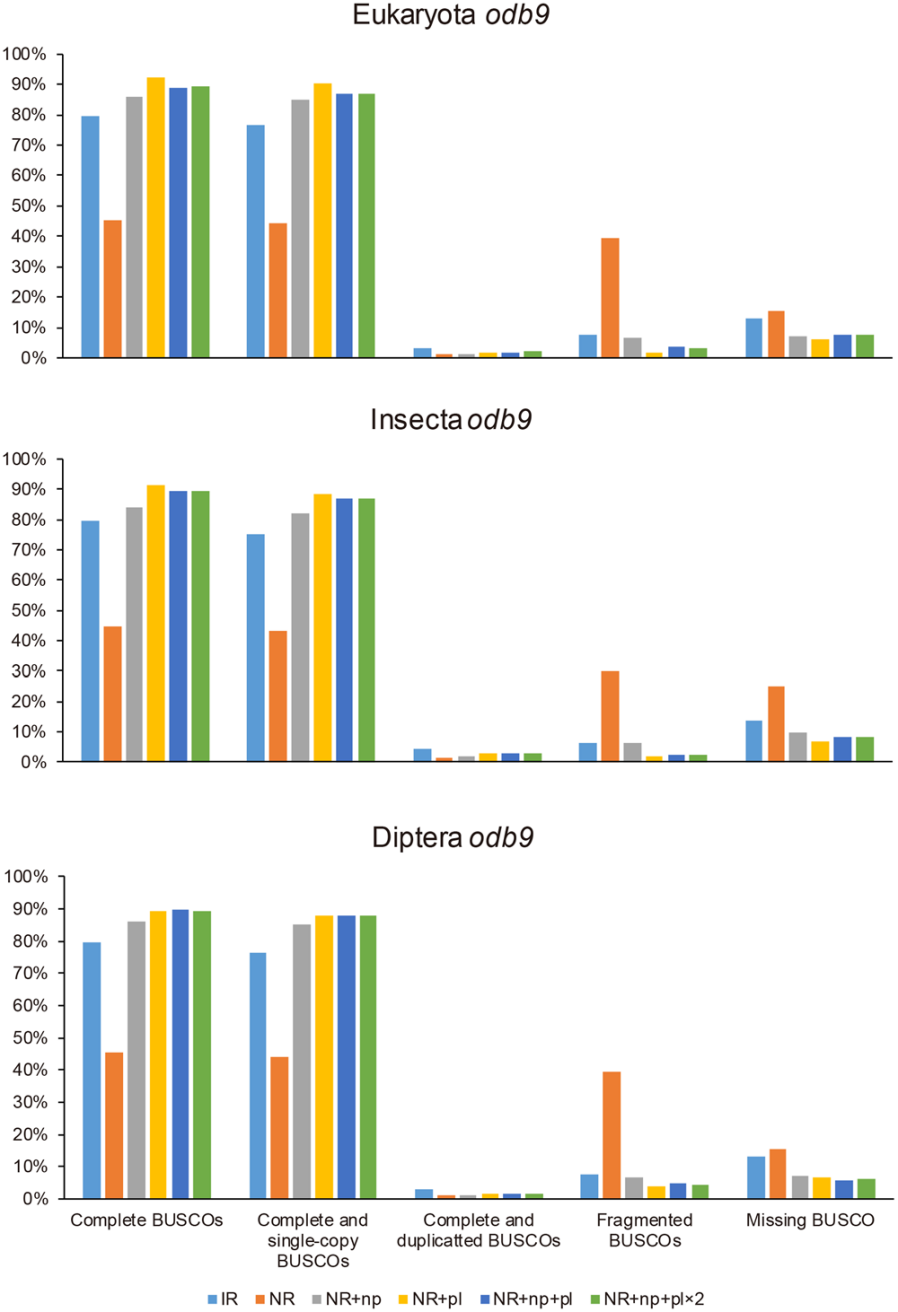
Gene set completeness of predicted gene model of draft genome sequences using benchmarking universal single-copy orthologs (BUSCO) analysis. The gene set completeness of the six draft genome sequences was calculated using BUSCO against Eukaryota *odb9*, Insecta *odb9*, and Diptera *odb9*. Before genome polishing, the low-quality bases of the NR reduced the accuracy of prediction in the gene model through MAKER2. Therefore, the gene set completeness was reduced and there was an increase in the number of “Fragmented BUSCOSs” and “Missing BUSCOs.” Genome polishing of the NR improved the gene set completeness, and genome polishing using Illumina reads markedly improved genome polishing using signal-level data in the BUSCO analysis.

We performed a BUSCO analysis against three datasets (Eukaryota *odb9*, Insecta *odb9*, and Diptera *odb9*) to assess the annotated gene set completeness of the assemblies. In the NRs, the gene set completeness increased markedly after genome polishing (Fig. 4 and Table 9). The gene set completeness of NR + np exceeded that of the IR. Genome polishing using Pilon (NR + pl, NR + np + pl, and NR + np + pl × 2) improved the gene set completeness by more than 88.8% against Eukaryota *odb9*, by 89.5% against Insecta *odb9*, and by 84.2% against Diptera *odb9*, irrespective of genome polishing using Nanopolish or the number of Pilon repetitions. Before genome polishing, the NR had low gene set completeness (below 50%). Fragmented BUSCOs appeared to increase owing to their low accuracy in the assembly (Fig. 4 and Table 9). The IR had a gene set completeness of 79.5% against Eukaryota *odb9*, 79.7% against Insecta *odb9*, and 67.8% against Diptera *odb9*.

**Table 9:** BUSCO completeness assessments for gene sets.

Database	Assemblies and genome polishing	Complete BUSCOs	Duplicated BUSCOs	Fragmented BUSCOs	Missing BUSCOs	Total BUSCO groups searched orthologs
Eukaryota odb9	IR	79.5%	3.0%	7.6%	12.9%	303
	NR	45.2%	1.0%	39.6%	15.2%	303
	NR + np	86.1%	1.0%	6.6%	7.3%	303
	NR + pl	89.4%	1.7%	4.0%	6.6%	303
	**NR + np + pl**	**89.8%**	**1.7%**	**4.6%**	**5.6%**	303
	NR + np + pl × 2	89.4%	1.7%	4.3%	6.3%	303
Insecta odb9	IR	79.7%	4.5%	6.4%	13.9%	1,658
	NR	44.8%	1.6%	30.1%	25.1%	1,658
	NR + np	84.1%	1.9%	6.2%	9.7%	1,658
	NR + pl	89.5%	2.5%	3.2%	7.3%	1,658
	**NR + np + pl**	**90.8%**	**2.6%**	**3.0%**	**6.2%**	1,658
	NR + np + pl × 2	90.0%	2.6%	3.0%	6.9%	1,658
Diptera odb9	IR	67.8%	3.5%	13.0%	16.3%	2,799
	NR	25.2%	0.6%	24.6%	50.2%	2,799
	NR + np	73.1%	1.7%	13.2%	13.7%	2,799
	NR + pl	83.6%	2.6%	8.4%	8.0%	2,799
	**NR + np + pl**	**84.0%**	**2.5%**	**8.5%**	**7.6%**	2,799
	NR + np + pl × 2	83.9%	2.4%	8.1%	8.0%	2,799

IR = the draft genome sequence assembled from the Illumina reads; NR = the draft genome sequence assembled from nanopore reads. The bold characters indicate the best statistics of gene sets completeness using BUSCO.

## Conclusion

Recently, reports of genome assemblies produced from nanopore reads have increased, and the improvement to contiguity in such genome assemblies is seen as a benefit of using long reads^8^. Therefore, we applied nanopore reads to a draft genome of *P. steinenii* assembled from Illumina MiSeq data, and investigated the difference in annotation. Low-quality nanopore reads were sufficient to improve the genome completeness, but nanopore reads alone were not sufficient to improve the annotation quality of the assembly when compared with that of the draft assembly produced using Illumina reads. Genome polishing with high-quality reads effectively improved the gene set completeness of the genome assembly produced using nanopore reads. Through MAKER annotation, we could identified the improvements in the gene set completeness without a difference in AED value. The genome of *P. steinenii* is smaller than 150 Mbp, so just one MinION cell is sufficient to increase the quality of its assembly and annotation.

## Materials and Methods

### Sample and DNA preparation

We collected *P. steinenii* adults from fresh water on King George Island, West Antarctica (62° 14′ S, 58° 47′ W) during 2018. We used 50 adult midges for DNA preparation. Genomic DNA was extracted using a DNeasy Tissue Kit (Qiagen, Valencia, CA, USA), and we used 2 μg of DNA for library construction and sequencing.

### Oxford Nanopore Technology library preparation and 1D sequencing

We constructed a genomic library for ONT sequencing using the ONT 1D ligation sequencing kit (SQK-LSK108) according to the manufacturer’s instructions^8,9^. We constructed the library in three steps and measured the DNA concentration using a PicoGreen assay at the end of each step (Table 1). First, we subjected 2.0 μg of genomic midge DNA to DNA repair using an NEBNext FFPE Repair Mix (NEB cat no. M6630) to eliminate DNA fragmentation. After purification using AMPure XP beads, we subjected the repaired genomic DNA to end repair and dA-tailing using an NEBNext Ultra II End-Repair/dA-tailing Module (NEB cat no. E7546), and purified the DNA using AMPure XP beads. We ligated an adapter for sequencing to the purified DNA using adapter mix 1D in an SQK-LSK108 kit and an NEB Blunt/TA ligase Master Mix (NEB cat no. M0367). Finally, we cleaned-up the adaptor-ligated DNA using AMPure XP beads, an ABB buffer, and an elution buffer. We quantified the final library using a Qubit.

### Oxford nanopore technology library preparation and 1D sequencing

We carried out sequencing using a GridION X5 sequencer and a single 1D flow cell (FLO-MIN106) with protein pore R9.4 1D chemistry for 48 h according to the manufacturer’s instructions. The FAST5 files generated during sequencing were live base-called using Guppy software (ver. 0.5.1) installed on GridION X5 using the default parameters. Sequencing and base-calling were controlled using ONT MinKNOW software (ver. 1.14.1). The FASTQ files obtained by base-calling were merged into single files and used for trimming using Porechop (ver. 0.2.3)^22^. All sequencing procedures were performed by Phyzen Co. Ltd. (Seongnam, Korea).

### *De novo* genome assembly of Illumina reads

The sequencing reads generated from the paired-end library (400 bp: SRX1976250) and the mate-pair library (3 kbp: SRX1976251 and 5 kbp: SRX1976252) from a previous study^5^ were trimmed using fastq_quality_trimmer in the FASTX-Toolkit (ver. 0.0.11)^23^ with the parameters “-t 30 –l 200 –Q 33”, and the resulting trimmed Illumina reads were assembled into scaffolds using ALLPATHS-LG (ver. 44849)^24^. The resulting scaffold sequence contained information about ambiguities within the assembly. These ambiguities are also represented as a comma-separated list of alternatives within curly braces in extended FASTA (eFASTA) format, which is another output format in ALLPATHS-LG. We removed the assembly ambiguity information using the efasta2fasta script^25^, which converts eFASTA to FASTA. The gaps in the resulting scaffolds were filled using GapFiller (ver. 2.1.1) with the parameters “-m 30 -o 2 -r 0.7 -n 5 -d 3000 -t 5 -g 1 -T 10 -i 1”^26^.

### Error correction and *de novo* genome assembly of nanopore reads

*De novo* genome assembly was performed using Canu-SMARTdenovo methods^15^. Nanopore reads were corrected using Canu (ver. 1.1.1)^16^. As the default parameters of Canu are applicable to a single 1D flow cell with protein pore R9.4 1D chemistry, and the genome size of *P. steinenii* predicted with GenomeScope is 143.8 Mbp according to a previous study^5,27^, we corrected the trimmed reads with default parameters and with “genomeSize = 140 m –nanopore-raw” according to Canu FAQ^28^. The resulting reads were assembled using SMARTdenovo^15,17^. A dot matrix over-lapper was selected as the over-lapper engine, and k-mer was set to 16.

### Genome polishing and the identity values of the draft genome sequences

We aligned sequencing reads obtained from ONT using Burrows-Wheeler Aligner (BWA; ver. 0.7.17)^18^ with parameters “-x ont2d”, and these were polished using Nanopolish (ver. 0.10.1)^10^. MiSeq reads were also aligned using BWA, and the obtained information was used for genome polishing using Pilon (ver. 1.22)^14^. The identity values of the draft genome sequence assembled from nanopore reads were computed based on the draft genome sequence assembled from the Illumina reads using the nucmer command in the MUMmer tool (ver. 3.0.) with parameters “-l 100 –c 500 –maxmatch”^8,29^. The resulting delta file was processed with the dnadiff script in the MUMmer tool, and average 1-to-1 alignment identity was used^8^.

### Repeat analysis and non-coding RNA

Repeat sequences for *P. steinenii* were predicted using RepeatMasker (ver. 3.3.0)^30^, a *de novo* repeat library was used as the database, and rmblastn (ver. 2.6.0) was used as a search program^31^. A *de novo* repeat library was constructed using RepeatModeler (ver. 1.0.11)^32^, including the RECON (ver. 1.08)^32^ and RepeatScout (ver. 1.0.5) software^33^, with default parameters. Tandem repeats, including simple repeats, satellites, and low-complexity repeats, were predicted using TRF^34^. Putative tRNA genes were identified using tRNAscan-SE (ver. 2.0)^35^ with option “-E -H”.

### Gene annotation

We carried out gene annotation using the MAKER annotation pipeline^21,36^. We used the RepBase library (ver. 20170100)^37^ to mask the repeat sequence in the draft genome with RepeatMasker (ver. 3.3.0)^30^, and selected the SNAP gene finder^38^ for *ab initio* gene prediction. RNA and protein sequences used in previous studies were aligned and used to find the best possible gene model in MAKER2^36^. Upper limit of the AED metric for controlling the quality of annotation for the final gene predictions was set to 1 in MAKER2^36^.

### Genome and gene set completeness of draft genome sequences

The genome completeness and gene set completeness of the draft genome sequences was validated using BUSCO (ver. 3)^19,20^. For the Augustus step in BUSCO, training data set for *Aedes aegypti* was selected. We conducted BUSCO analyses against Eukaryota, Insecta, and Diptera datasets.

### Accession codes

The raw data have been deposited at the National Center for Biotechnology Information (NCBI) BioProject repository PRJNA284858 (SRX5001002).
